# Same gene, opposite sexes: Sex-specific divergent expression of a gene required for vertebrate fertilization

**DOI:** 10.1073/pnas.2116001118

**Published:** 2021-10-12

**Authors:** Enrica Bianchi

**Affiliations:** ^a^Cell Surface Signalling Laboratory, Wellcome Sanger Institute, Cambridge CB10 1SA, United Kingdom

The vast majority of eukaryotes reproduce sexually; each species generates highly specialized cells with distinct morphological and functional characteristics that differ depending on the individual’s genetic sex. The male spermatozoon (sperm) is generally a motile cell that bears the paternal genome (1, 2), while the female oocyte usually is a large, immotile cell that stores the maternal genome together with all the molecules required to initiate the development of the new organism after fertilization (3).

Sexual reproduction relies on fertilization—the process whereby sperm and eggs fuse and pass the genetic information to the next generation, thereby creating diverse, genetically unique organisms. Furthermore, fertilization marks the end of the gametes’ lives as individual cells and triggers a plethora of molecular events aiming at the development of a new individual.

In PNAS Fujihara et al. (4) report that a cell-surface protein conserved across vertebrates is required for fertilization, and they show that its expression is restricted to the male or to the female gametes in the mouse and zebrafish, respectively, and consequently that its mode of action differs in mammals and fish.

The eggs of all vertebrates are surrounded by extracellular coats that initially protect the female gamete and then the conceptus during embryonic development (5). The site of synthesis, biophysical features, and molecular composition of the egg vestments vary depending on the species, but in every one the innermost layer maintains a conserved network structure made of interconnected fibrils formed by several homologous proteins. This protective matrix is known as the chorion in the modern bony fishes (teleosts) like the zebrafish, the zona pellucida (ZP) in mammals, and the vitelline envelope in amphibians and birds. In particular, the fibrils are made of three glycoproteins in mice and four in humans (6), while their number varies greatly in other species (7, 8), and the two major components of the zebrafish chorion are the proteins Zp2 and Zp3 (9, 10).

The ZP represents the last hurdle for the sperm before the egg membrane can be reached, but while mammalian sperm pass through the extracellular matrix, the chorion of zebrafish is provided with a minute opening—the micropyle—where the sperm can bind directly to the egg membrane and finally fuse (11). In mammals, our understanding of the mechanism driving the sperm through the ZP is still incomplete. Before fertilization, the sperm undergoes a significant structural rearrangement known as the acrosome reaction that is necessary for the sperm to be competent to fertilize the egg. The acrosome is a large vesicle localized underneath the plasma membrane in the sperm head and overlying the nucleus. During the acrosome reaction the contents of the vesicle are released, and membrane proteins sequestered within the acrosome are exposed on the sperm head. The stimuli that trigger the acrosome reaction are still debated, and it is currently unclear whether the mammalian sperm display a receptor capable of binding the ZP proteins or the glycans decorating them (12, 13).

Following the passage of the ZP the cell membranes of sperm and egg bind and fuse to complete fertilization. The first-ever-identified pair of proteins essential for sperm–egg binding in mammals were IZUMO1 on the sperm (14) and JUNO on the egg (15) and, so far, this remains the only pair we know. Recently, more proteins displayed on the sperm were shown to be required for fertilization, and their identification is shedding light on the mysterious mechanism that underlies sperm–egg interaction (16–19). Nonetheless , it is unclear whether they have a binding partner on the egg membrane like IZUMO1, how they cooperate, or if they are involved in the formation of multimolecular complexes (20).

## The Discovery of Bouncer

In 2018 Pauli and coworkers (21) showed that a glycosylphosphatidylinositol-anchored protein, aptly named Bouncer, is essential for fertilization in fish and particularly that it is required for the interaction of sperm with the egg membrane. To investigate the role of this single-exon gene, they generated knockout fish of the species *Danio rerio*, commonly known as the zebrafish, using CRISPR genome editing technology. Bouncer is expressed in fish oocytes and has homologs in all vertebrate species. The oocytes of fish lacking Bouncer were unable to be fertilized by wild-type sperm, demonstrating that the protein is essential for fertilization. Moreover, the authors showed that swapping Bouncer genes between two distant species of fish, zebrafish and medaka, was sufficient to obtain hybrid individuals. Together, these results indicated that Bouncer is essential for fertilization and sufficient to determine species specificity in fish (21). It also pointed to a direct role of Bouncer during the sperm–egg binding/fusion step.

**Fig. 1.**
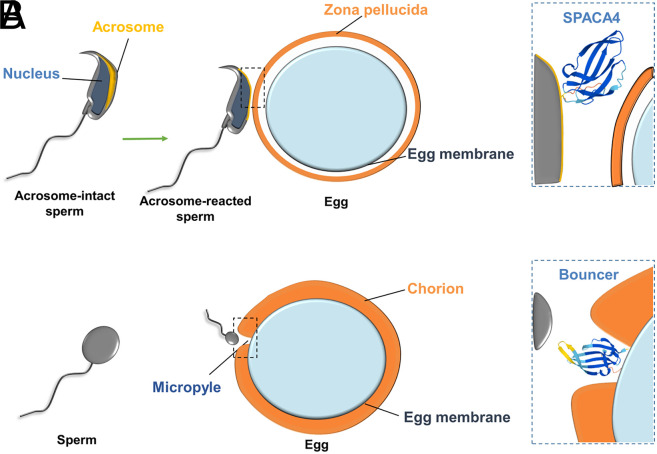
The homologous proteins SPACA4 and Bouncer have sex-specific expression patterns and different mechanisms of action. (*A*) SPACA4 is displayed on the surface of the mouse sperm head after the acrosome reaction and is required for binding/passing the ZP. (*B*) In zebrafish, Bouncer is expressed in the egg and is required for binding/fusion of sperm and egg cell membranes. The three-dimensional protein structure model was obtained from the AlphaFold Protein Structure Database (https://www.alphafold.ebi.ac.uk/). The regions with the highest model confidence are in blue. Sperm and eggs are not drawn to scale.

In their paper in PNAS, Fujihara et al. (4) set out to elucidate the role of Bouncer’s homolog in mammals. They show using evolutionary analysis that SPACA4 (Sperm Acrosome Associated 4) and Bouncer share some conserved features such as the small number of amino acids, the number and distribution of cysteines, and a particular folding characteristic of proteins belonging to the Ly6/uPAR family. Remarkably, while Bouncer is displayed on the cell surface of eggs in fish, the expression of *Spaca4* is restricted to the male germ cells in mammals (4), in which the protein localizes within the membrane of the sperm acrosome and is only exposed on the sperm head after the acrosome reaction. Therefore, Bouncer and SPACA4 represent an interesting example of homologous proteins with divergent and sex-specific expression patterns. The reasons and mechanisms that drove this evolution need to be further investigated. To elucidate the functional role of SPACA4, the authors generated knockout mice and assessed the fertilizing ability of sperm lacking SPACA4. Differently from what happens in zebrafish, mouse sperm bind and fuse normally to eggs that are freed from the ZP even in the absence of SPACA4, but they are unable to efficiently fertilize eggs with an intact ZP in the absence of SPACA4 (figure 3 in ref. 4). These results strongly point toward a role of the mammalian sperm protein SPACA4 in binding to and traversing the ZP (Fig. 1). One possible function of SPACA4 is to bind directly to molecules within the ZP; however, the biochemical characteristics of these interactions remain to be fully investigated. Interestingly, the protein was initially studied in human fertilization (22) and was shown to be involved in the interaction of sperm with the egg membrane. Whether the protein has evolved different roles among mammalian species also requires a thorough investigation.

The work of Fujihara et al. identifies a protein required for fertilization that represents an important link in the evolution of reproductive strategies in vertebrates.

The work of Fujihara et al. (4) identifies a protein required for fertilization that represents an important link in the evolution of reproductive strategies in vertebrates. Its varied expression pattern and modes of action between fish and mammals add another layer of complexity to the puzzle of fertilization. Our understanding of sperm–egg interaction has grown rapidly in recent years, but many questions remain unanswered. The most pressing ones can be summarized as follows (20):

1) How do sperm pass through the ZP, and which molecules are involved?
2) Are there other receptor pairs required for sperm binding to the egg membrane?
3) Which mechanism governs the final step of sperm–egg fusion? Does a molecule with fusogenic activity exist in mammalian gametes?

The limits imposed by intrinsic challenges like the paucity of biological material as well as the particular biochemical features of cell-surface interactions (23) could be overcome by technological advancements in sectors such as genetic manipulation, high-resolution microscopy, and single-cell technologies. Undoubtedly, the benefit of extending the investigation to more diverse species will be a better understanding of the process of fertilization.
